# Macrolide- and Rifampin-Resistant *Rhodococcus equi* on a Horse Breeding Farm, Kentucky, USA

**DOI:** 10.3201/eid1902.121210

**Published:** 2013-02

**Authors:** Alexandra J. Burton, Steeve Giguère, Tracy L. Sturgill, Londa J. Berghaus, Nathan M. Slovis, Jeremy L. Whitman, Court Levering, Kyle R. Kuskie, Noah D. Cohen

**Affiliations:** Author affiliations: University of Georgia, Athens, Georgia, USA (A.J. Burton, S. Giguère, T.L. Sturgill, L.J. Berghaus);; Hagyard Equine Medical Institute, Lexington, Kentucky, USA (N.M. Slovis);; Equine Medical Associates, Lexington (J.L. Whitman, C. Levering);; Texas A & M University, College Station, Texas, USA (K.R. Kuskie, N.D. Cohen)

**Keywords:** Rhodococcus equi, bacteria, horses, foals, equines, breeding farm, repetitive sequence–based PCR, macrolide resistance, rifampin resistance, antimicrobial drug resistance

## Abstract

Macrolide and rifampin resistance developed on a horse breeding farm after widespread use was instituted for treatment of subclinical pulmonary lesions in foals. Resistance occurred in 6 (24%) of 25 pretreatment and 8 (62%) of 13 (62%) posttreatment isolates from affected foals. Drug-resistant isolates formed 2 distinct genotypic clusters.

*Rhodococcus equi* is a major cause of pneumonia in young horses and a common opportunistic pathogen of immunocompromised humans (*1*). Over the past decade, control of *R. equi* infections at many horse farms to which the disease is endemic has relied on early detection of subclinical pulmonary disease by use of thoracic ultrasonography and initiation of treatment with antimicrobial drugs before development of clinical signs (*2*). This approach appears to have decreased deaths caused by *R. equi* pneumonia at some farms, although controlled studies are lacking (*2*). However, the temporal association between widespread use of macrolides and rifampin and a perceived increase in the frequency of detection of drug-resistant isolates in the past decade (*3*) suggest that this practice may not be innocuous.

We describe emergence of resistance to macrolides and rifampin among *R. equi* isolates obtained from a horse breeding farm. We conducted this study after initiation of an ultrasonographic screening program on the farm and resulting widespread use of these drugs in foals with subclinical pulmonary lesions.

## The Study

This study was conducted at a Thoroughbred horse breeding farm in Kentucky, USA. The farm initiated an ultrasonographic screening program in 2001 in an attempt to decrease deaths associated with pneumonia caused by *R. equi* through early diagnosis and treatment of foals with subclinical lesions. During March–July 2010, the farm reported 9 foals infected with macrolide- and rifampin-resistant *R. equi* isolates. This finding led to a disease investigation consisting of retrospective data collection (Table 1), collection of air samples in September 2010 to determine the prevalence of drug-resistant *R. equi* in the environment, and prospective culture of pulmonary lesions from all foals in 2011 before initiation of antimicrobial drug therapy.

**Table 1:** Macrolide- and rifampin-resistant *Rhodococcus equi* on horse breeding farm, Kentucky, USA*

Year	No. foals born	No. (%) foals with lesions treated	No. foals tested	No. foals with positive *R. equi* culture	No. (%) foals with macrolide- and rifampin-resistant *R. equi*
2001	95	30 (32)	30	30	0
2002	117	53 (45)	0	NA	NA
2003	148	58 (32)	2	?	?
2004	181	88 (49)	28	19	0
2005	168	70 (42)	30	?	?
2006	170	42 (41)	5	2	0
2007	181	93 (51)	4	?	?
2008	171	52 (30)	21	16	4 (25)†
2009	162	50 (31)	30	22	5 (23)†
2010	138	45 (33)	28	22	9 (41)†
2011	132	24 (18)	27	25	9 (36)†

*NA, not applicable; ?, data missing from farm records. †Includes pretreatment and posttreatment isolates.

A total of 124 air samples were collected from the 4 barns at the farm and from each of their respective surrounding paddocks by using a portable air-sampling device as described (*4*). For the 2011 breeding season, a tracheobronchial aspirate was collected transendoscopically from each foal that had ultrasonographically detected pulmonary lesions before initiation of therapy by using a triple-guarded microbiological aspiration catheter. A second tracheobronchial aspirate was collected by using the same method 2 weeks after initiation of therapy. Decisions regarding the need for therapy and selection of antimicrobial agents were made by the farm veterinarian and manage.

After standard microbiological culture of air and tracheobronchial aspirate samples, confirmation of *R. equi* was accomplished by amplification of the *choE* gene and detection of the virulence plasmid by amplification of the *vapA* gene by using multiplex PCR (*5*). For each isolate, the MICs of erythromycin, azithromycin, clarithromycin, and rifampin were determined from 3–5 isolated colonies by broth dilution in accordance with Clinical and Laboratory Standards Institute guidelines (*6*). *R. equi* isolates with MIC values <2 µg/mL for azithromycin and clarithromycin, <0.5 µg/mL for erythromycin, and <1 µg/mL for rifampin were considered susceptible, and isolates with MIC values >8 µg/mL were considered resistant.

The similarity of *R. equi* isolates from air and tracheobronchial aspirate samples was determined by using a repetitive sequence–based PCR (DiversiLab; bioMérieux Inc., Durham, NC, USA) previously validated for *R. equi* (*7*). Isolates were clustered as the same strain on the basis of >95% similarity and a difference of <1 band (*8*).

The ultrasonographic screening program was introduced in 2001, and the first isolates of *R. equi* resistant to macrolides and rifampin were identified in 2008 (Table 1). There were no *R. equi* isolates that were resistant to either a macrolide or rifampin alone. Air sampling yielded 82 isolates of *R. equi*. Of these isolates, 15 (18%) contained the plasmid required for virulence in foals and 67 (82%) were avirulent.

All 15 virulent isolates and 23 randomly selected avirulent isolates were used for in vitro antimicrobial drug susceptibility testing and genotyping. Two (5%) of 38 isolates tested were resistant to azithromycin, clarithromycin, erythromycin, and rifampin. One resistant isolate was virulent and came from an outdoor paddock location that had been used to house foals given a diagnosis of *R. equi* pneumonia caused by drug-resistant isolates. The other resistant isolate was avirulent and was isolated from an air sample obtained from an indoor barn location.

During the 2011 season, 132 foals were born on the farm. Thoracic ultrasonography showed evidence of pulmonary disease in 27 (20%) foals. Culture of a tracheobronchial aspirate before initiation of therapy yielded *R. equi* in 25 (93%) of the 27 foals sampled (Table 1). Of the 25 pretreatment *R. equi* isolates, 6 (24%) were resistant to macrolides and rifampin. Twenty-four foals were treated with clarithromycin and rifampin. Tracheobronchial aspirates were collected 2 weeks after initiation of therapy in 19 foals, and *R. equi* was cultured from 13 (68%) of the 19 samples. Of these 13 posttreatment isolates, 8 (62%) were resistant to macrolides and rifampin. After identification of resistant isolates, 3 foals had a third antimicrobial agent added to their therapy (gentamicin, n = 2; doxycycline, n = 1). All foals with a diagnosis of pneumonia caused by *R. equi* in 2011 survived.

**Figure 1:**
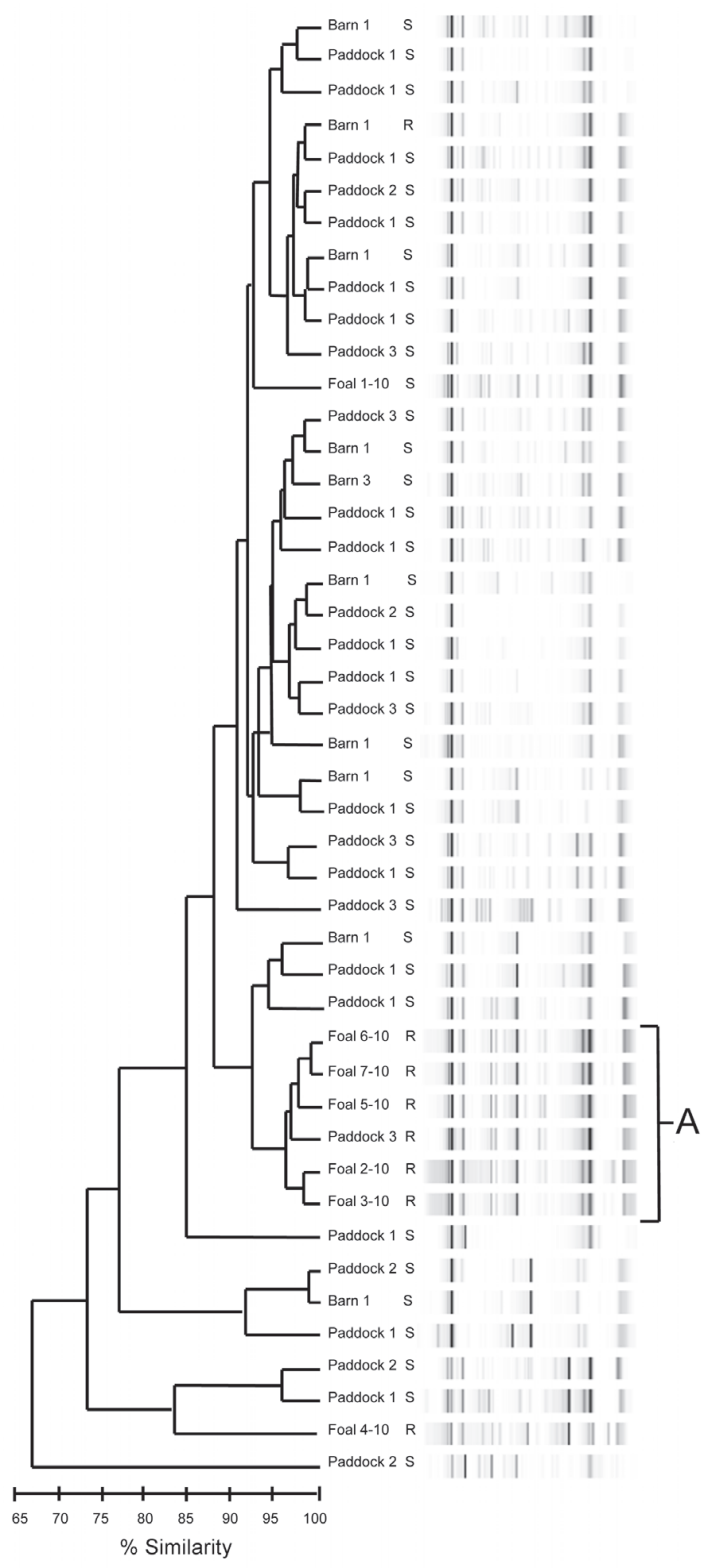
Dendrogram and virtual gel repetitive sequence–based PCR fingerprint patterns of foal and air (barn and paddock)–derived isolates of *Rhodococcus equi* on horse breeding farm, Kentucky, USA, 2010. Macrolide and rifampin susceptibility (S) or resistance (R) are indicated. A indicates the main cluster of drug-resistant isolates (5 foal and 1 air).

**Figure 2:**
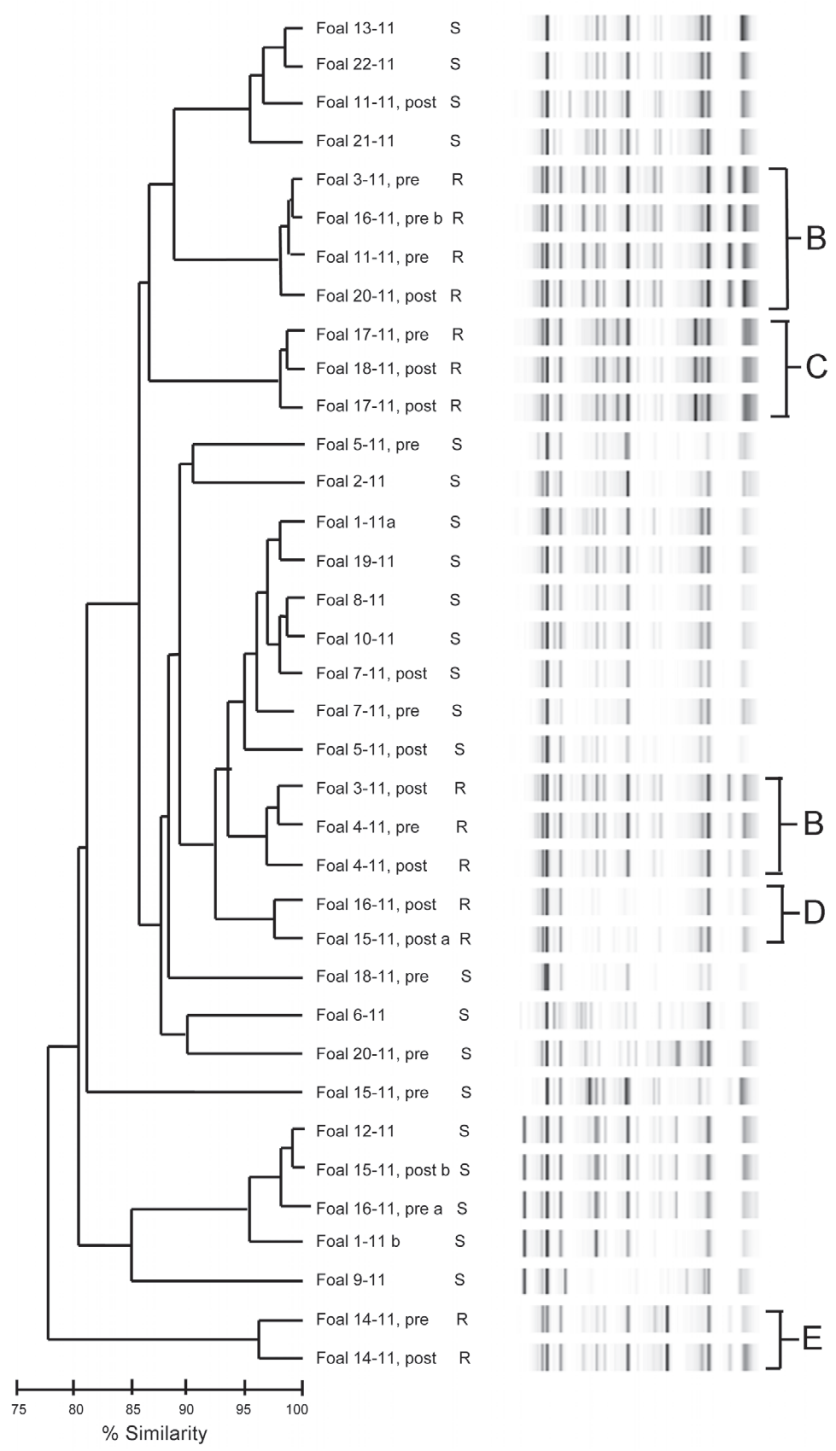
Dendrogram and virtual gel repetitive sequence–based PCR fingerprint patterns of 36 *Rhodococcus equi* isolates obtained from foals on horse breeding farm, Kentucky, USA, 2011. Macrolide and rifampin susceptibility (S) and resistance (R) are indicated. B–E indicates clusters of drug-resistant isolates. Foals from which pretreatment (pre) and posttreatment (post) samples were obtained are indicated. a and b indicate samples from which 2 isolates were obtained.

Genotypes by repetitive sequence–based PCR from all 2010 isolates grouped into 1 main cluster consisting of resistant isolates from 5 foals and 1 air sample (Figure 1, cluster A). Isolates resistant to macrolides and rifampin from the 2011 foals grouped closely, forming 4 clusters (B–E); the largest cluster (B) contained 7 isolates (Figure 2). Of the 11 foals for which pretreatment and posttreatment samples were collected in 2011, a total of 5 had similar pretreatment and posttreatment isolates and 6 had different pretreatment and posttreatment isolates (Table 2).

**Table 2:** Characteristics of pretreatment and posttreatment *Rhodococcus equi* isolates obtained from foals on a horse breeding farm, Kentucky, USA, 2011*

Foal ID no.	Pretreatment isolates (cluster)	% Similarity†	Posttreatment isolates (cluster)
7-11	S	97.5	S
5-11	S	84.3	S
14-11	R (E)	96.4	R (E)
3-11	R (B)	96.5	R (B)
4-11	R (B)	97.4	R (B)
17-11	R (C)	98.5	R (C)
20-11	S	83.1	R (B)
18-11	S	78	R (C)
11-11	R (B)	87.2	S
15-11‡	S	83.1	S
	NA	73.2	R (D)
16-11‡	S	78.8	R (D)
	R (B)	80.8	NA

*ID, identification; S, susceptible; R, resistant; NA, not applicable. †Determined by using Pearson correlation coefficient and unweighted pair-group method with arithmetic means. ‡Two isolates were recovered from the pretreatment (foal ID 16-11) or posttreatment (foal ID 15-11) sampling.

## Conclusions

Recently, development of macrolide resistance in clinically relevant pathogens has been recognized in human and animal populations that received intensive macrolide treatment (*9**,**10*). We documented that macrolide- and rifampin-resistant isolates of *R. equi* occurred 7 years after initiation of an ultrasonographic screening program, which resulted in treatment of all foals with subclinical pulmonary lesions.

Compared with macrolide-susceptible *Campylobacter jejuni*, acquisition of macrolide resistance impairs the fitness and transmission of the pathogen in chickens, suggesting that the prevalence of macrolide-resistant *C. jejuni* would probably decrease in the absence of antimicrobial drug selection pressure (*11*). Similarly, studies in humans have shown that macrolide resistance in *Streptococcus pneumoniae* decreased 2–5 years after use of azithromycin was stopped and selection pressure was abolished (*10*). Although the fitness cost of macrolide resistance among *R. equi* isolates might be sufficient to ensure its eventual elimination, this elimination will take time and elimination of resistance will only occur in the absence of antimicrobial drug selection pressure (*10*).

A recent study on a large horse farm indicated that the proportion of foals with ultrasonographic pulmonary lesions associated with *R. equi* infection that recovered was not different between foals given azithromycin and rifampin and foals given a placebo (*12*). This surprising finding, combined with the apparent increase in macrolide- and rifampin-resistance demonstrated in the present study, support the need to stop the practice of mass macrolide treatment for subclinical infection with *R. equi* in foals on horse breeding farms. The goal should be to more accurately identify, of the many subclinically infected foals, which few are likely to show development of disease and thus require treatment for it.
